# Supplementary material for: Active site specificity profiling datasets of matrix metalloproteinases (MMPs) 1, 2, 3, 7, 8, 9, 12, 13 and 14
Source: Data Brief. 2016 Feb 22;7:299–310. doi: 10.1016/j.dib.2016.02.036 (PMC4777984; doi:10.1016/j.dib.2016.02.036)
Supplement: Supplementary file 10 — Supplementary material [file mmc10.zip › WebPICS_hMMP14_G_1%/P1.html]

 

PICS results


|  |  |
| --- | --- |
| **P1\_A**  33 in 213 sites   15.5 %    effects > 10 perc. pnts.  (vice-versa in brackets)  P3\_P: 14.6 (17.8)   P2\_K: 17.9 (29.5)   P2prime\_K: 18.0 (11.4) |  |
| **P1\_C**  6 in 213 sites   2.8 %    effects > 10 perc. pnts.  (vice-versa in brackets)  P2prime\_M: 13.4 (11.5) |  |
| **P1\_G**  38 in 213 sites   17.8 %    effects > 10 perc. pnts.  (vice-versa in brackets)  P3prime\_V: 14.6 (22.2) |  |
| **P1\_H**  11 in 213 sites   5.2 %    effects > 10 perc. pnts.  (vice-versa in brackets)  P3\_C: 15.4 (28.1)   P2prime\_N: 13.5 (14.8)   P3prime\_K: 34.2 (15.6) |  |
| **P1\_N**  28 in 213 sites   13.1 %    effects > 10 perc. pnts.  (vice-versa in brackets)  P3\_I: 21.1 (36.9) |  |
| **P1\_P**  11 in 213 sites   5.2 %    effects > 10 perc. pnts.  (vice-versa in brackets)  P2prime\_M: 14.9 (23.4) |  |
| **P1\_S**  23 in 213 sites   10.8 %    effects > 10 perc. pnts.  (vice-versa in brackets)  P2\_S: 21.0 (24.2) |  |
| **P1\_W**  4 in 213 sites   1.9 %    effects > 10 perc. pnts.  (vice-versa in brackets)  P2\_N: 42.5 (10.6) |  |
| **P1\_Y**  9 in 213 sites   4.2 %    effects > 10 perc. pnts.  (vice-versa in brackets)  P2\_Q: 28.1 (23.1)   P3prime\_M: 30.0 (38.7)   P3prime\_Q: 27.7 (20.8) |  |
